# Simulated Estimates of Pre-Pregnancy and Gestational Diabetes Mellitus in the US: 1980 to 2008

**DOI:** 10.1371/journal.pone.0073437

**Published:** 2013-09-05

**Authors:** Maria E. Mayorga, Odette S. Reifsnider, David M. Neyens, Mulugeta G. Gebregziabher, Kelly J. Hunt

**Affiliations:** 1 Department of Industrial & Systems Engineering, North Carolina State University, Raleigh, North Carolina, United States of America; 2 Department of Industrial Engineering, Clemson University, Clemson, South Carolina, United States of America; 3 Department of Public Health Sciences, Medical University of South Carolina, Charleston, South Carolina, United States of America; College of Pharmacy, University of Florida, United States of America

## Abstract

**Purpose:**

To simulate national estimates of prepregnancy and gestational diabetes mellitus (GDM) in non-Hispanic white (NHW) and non-Hispanic black (NHB) women.

**Methods:**

Prepregnancy diabetes and GDM were estimated as a function of age, race/ethnicity, and body mass index (BMI) using South Carolina live singleton births from 2004–2008. Diabetes risk was applied to a simulated population. Age, natality and BMI were assigned to women according to race- and age-specific US Census, Natality and National Health and Nutrition Examination Surveys (NHANES) data, respectively.

**Results:**

From 1980–2008, estimated GDM prevalence increased from 4.11% to 6.80% [2.68% (95% CI 2.58%–2.78%)] and from 3.96% to 6.43% [2.47% (95% CI 2.39%–2.55%)] in NHW and NHB women, respectively. In NHW women prepregnancy diabetes prevalence increased 0.90% (95% CI 0.85%–0.95%) from 0.95% in 1980 to 1.85% in 2008. In NHB women from 1980 through 2008 estimated prepregnancy diabetes prevalence increased 1.51% (95% CI 1.44%–1.57%), from 1.66% to 3.16%.

**Conclusions:**

Racial disparities in diabetes prevalence during pregnancy appear to stem from a higher prevalence of prepregnancy diabetes, but not GDM, in NHB than NHW.

## Introduction

Several factors influence diabetes prevalence during pregnancy and thus, make it difficult to compare prevalence over time or across populations. Namely, changes in diagnostic criteria as well as screening policies concerning gestational diabetes mellitus (GDM). At the 4^th^ International Workshop conference on GDM in 1997, a critical change in the GDM diagnostic criteria occurred when it was largely agreed that the Carter & Coustan criteria replace the National Diabetes Data Group criteria, significantly lowering the accepted cut-points, thus increasing the prevalence of GDM [1]. Moreover, as awareness of GDM has increased screening has also increased, further increasing the prevalence of diagnosed GDM. Finally, because GDM encompasses undiagnosed type 2 diabetes prior to pregnancy, the definition, screening strategies, and awareness of type 2 diabetes ultimately influences the observed prevalence of GDM. The diagnostic criteria and screening practices for diabetes changed in 1997 when the threshold for a fasting glucose level diagnostic of diabetes was lowered from 140 mg/dL to 126 mg/dL (7.0 to 7.8 mmol per L) [2], [3].

Increased maternal age and prepregnancy BMI are both strong risk factors for GDM and contributing factors to increased prevalence rates. However, using standard methodology it is not feasible to examine the impact of these factors on GDM prevalence without systematically following a large cohort of women. As pregnancy is a rare event, in countries without national registries, such as the US, it is not feasible to routinely obtain a nationally representative sample of pregnant women and complete a clinical examination during pregnancy. Tracking members through their electronic medical records as has been done in the Kaiser managed health care organization comes the closest to a comprehensive cohort [4], but remains subject to secular trends in the diagnosis of GDM and type 2 diabetes (i.e., changes in the diagnostic criteria as well as changes in screening strategies and awareness of type 2 diabetes in women of childbearing age).

Estimates of recognized gestational diabetes during pregnancy from prior studies range from 2 to 10% of the pregnancies in the US, with higher estimates for racial and ethnic minority groups than for non-Hispanic whites (NHWs) [5]. Once diagnosed with GDM, a woman has a high chance of developing type 2 diabetes, with cumulative incidence estimates of 15–50% in the decades following delivery [6]. A recent meta-analysis reports a 7-fold increase in risk of developing type 2 diabetes in women with prior GDM relative to women without diabetes during pregnancy [7]. Other studies indicate that women with GDM also have increased cardio-metabolic and cardiovascular disease risk[7]–[9]. Moreover, the ‘early origin of disease’ hypothesis proposes that gestational programming may critically influence adult health and disease [10]. Exposure to maternal diabetes early in pregnancy is associated with fetal loss, perinatal mortality and birth defects [11]. Exposure to maternal diabetes later in pregnancy has been associated with high birthweight, macrosomia, increased childhood and adult obesity and increased risk of type 2 diabetes[12]–[18]. We postulate that if the diabetic intrauterine environment substantially contributes to the obesity and diabetes epidemics, their prevalence will continue to increase perpetuating health disparities between racial and ethnic groups, as populations with high prevalence will continue to be disproportionately exposed.

Previous studies have focused on projections of diabetes prevalence in the general population, and have not included GDM. Projections of diabetes prevalence in the US population have been completed through 2030 [19] and 2050 [20] and also globally through 2030 [21]. In contrast, our objective was to estimate the prevalence of prepregnancy diabetes and GDM in the US from 1980 through 2008 in NHW and NHB women 15 to 44-years-old. Additionally, we investigated the extent that the obesity epidemic and maternal age at delivery have influenced trends in diabetes during pregnancy.

## Methods

**Figure 1:**
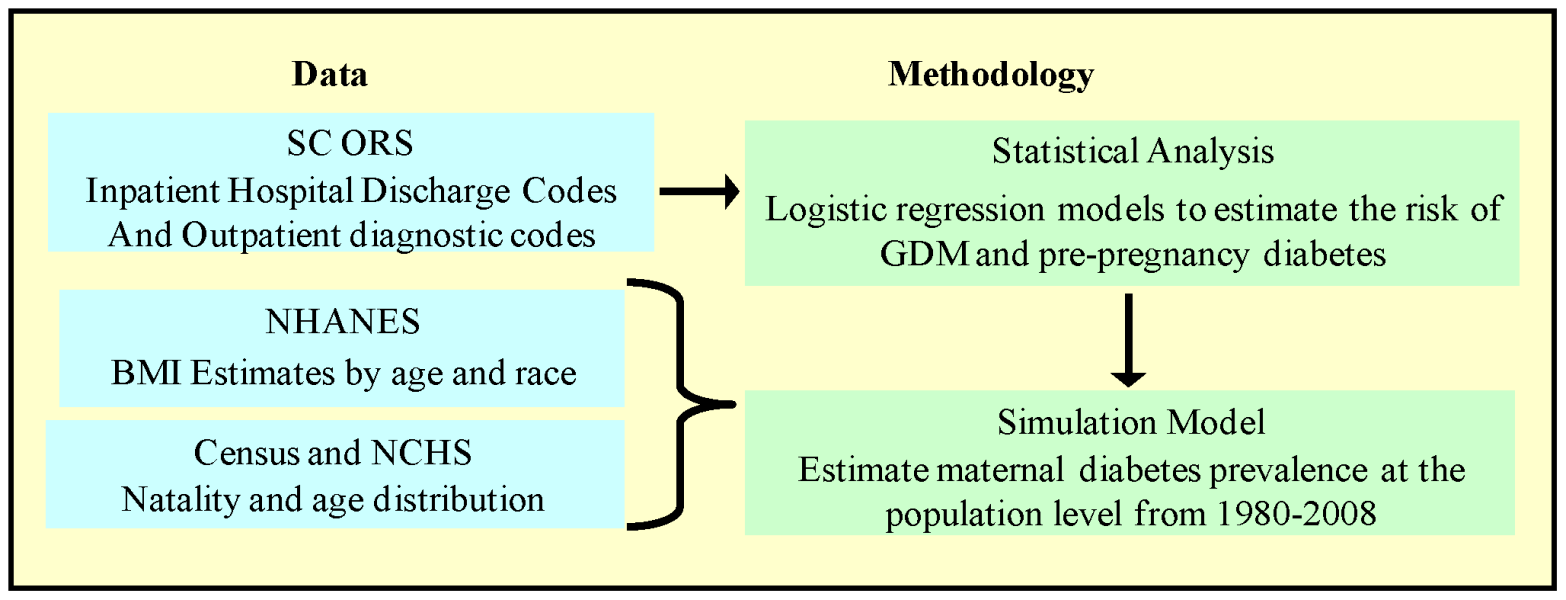
High level overview of methodology. SC ORS, SC Office of Research Statistics; NCHS, National Center for Health Statistics.

A mixed-methods approach was used with a statistical model used to inform the parameters of a simulation model. Figure 1 provides a conceptual representation of the methodology. In a logistic regression model, we estimate the risk of diabetes during pregnancy (prepregnancy and GDM) based on age, race/ethnicity, and body mass index (BMI). Then, we simulated a cohort with US population level characteristics (race/ethnicity, age, race- and age-specific BMI, and natality rates) and apply risk of diabetes based on regression estimates.

### Data

Live singleton births of South Carolina (SC) resident mothers between January 2004 and December 2008 were used to estimate the risk of diabetes during pregnancy. The mothers included in the study were 15 to 44-year-old NHW (n = 151,362) or NHB (n = 91,737) women. Birth certificate information was obtained from the SC Department of Health and Environmental Control and linked by the SC Office of Research and Statistics (ORS) to inpatient hospital discharge records to obtain maternal inpatient procedure and diagnostic codes pertaining to delivery. Additionally, outpatient diagnostic codes were available for the prenatal period if care was received through Medicaid or the State Health Plan. The algorithm linking the databases used personal identifying information and was developed by ORS.

US population level maternal diabetes prevalence projections were made using National Health and Nutrition Examination Surveys (NHANES) data on BMI and natality produced by the National Center for Health Statistics and age distribution data from the US Census. We used NHANES II (1976–1980), III (1988–1994), and continuous NHANES cycles 1999–2000, 2001–2002, 2005–2006, and 2007–2008 data [12]. Age- and race/ethnicity-specific natality data [22] and race-specific age distribution of NHW and NHB women ages 15–44[23]–[25] were obtained for 1980, 1990, 2000 and 2008. Table 1 provides extracted data.

**Table 1:** Characteristics of US NHW and NHB women ages 15–44.

A. US Census Age Distribution (%)								
	Age Group							
	15–19	20–24	25–29	30–34	35–39		40–44	
NHW								
1980	19.4	20.0	18.5	17.0		13.6	11.4	
1990	14.2	15.6	18.0	18.9		17.5	15.9	
2000	15.5	14.4	15.0	16.5		19.1	19.5	
2008	16.8	16.6	16.7	15.4		16.8	17.7	
NHB								
1980	22.5	21.4	18.6	15.3		11.9	10.3	
1990	17.0	17.0	18.3	18.5		16.2	13.0	
2000	17.1	16.0	15.8	16.3		17.7	17.0	
2008	18.7	17.2	16.9	15.2		15.9	16.1	
**B. NHANES** a **Mean BMI (kg/m^2^)**								
	**Age Group**							
	**15–19**	**20–24**	**25–29**	**30–34**		**35–39**	**40–44**	**Overall**
NHW								
1980	21.9	22.7	23.5	24.4		24.8	25.1	23.5
1990	22.7	23.6	24.3	25.5		26.6	26.3	24.8
2000	23.6	26.1	26.7	26.7		27.4	27.7	25.9
2008	23.6	26.2	27.3	27.9		27.9	28.9	26.7
NHB								
1980	22.8	23.9	26.5	27.5		29.0	28.3	25.6
1990	24.7	26.2	27.2	29.0		29.4	30.6	27.8
2000	25.7	29.1	30.1	31.3		30.1	32.7	28.4
2008	26.7	30.0	30.7	31.4		32.1	30.8	29.3
**C. US Natality (births per 1,000 women)**								
	**Age Group**							
	**15–17**	**18–19**	**20–24**	**25–29**		**30–34**	**35–39**	**40–44**
NHW								
1980	25.5	73.2	111.1	113.8		61.2	18.8	3.5
1990	29.5	78.0	109.8	120.7		81.7	31.5	5.2
2000	23.3	72.3	106.6	116.7		94.6	40.2	7.9
2008	19.3	65.0	99.2	116.6		101.8	47.2	9.7
NHB								
1980	72.5	135.1	140.0	103.9		59.9	23.5	5.6
1990	82.3	152.9	160.2	115.5		68.7	28.1	5.5
2000	49.0	118.8	141.3	100.3		65.4	31.5	7.2
2008	35.2	105.6	132.3	107.2		75.6	37.0	8.9

Abbreviations: NHW, non-Hispanic white; NHB, non-Hispanic black; SC, South Carolina; US, United States; BMI, body mass index; NHANES, National Health and Nutrition Examination Surveys.

a BMI estimates are from NHANES II (1980), NHANES III (1990), continuous NHANES cycles 1 and 2 (2000), and continuous NHANES cycles 4 and 5 (2008).

### Ethics Statement

The Institutional Review Board of the Medical University of South Carolina approved the study as exempt research (HR Number 19410, August 25, 2009 to August 25, 2014) and waived the need for informed consent given the use of routinely collected de-identified patient data for this analysis.

### Statistical Model Variable Definition

Diabetes during pregnancy was defined by either GDM or prepregnancy diabetes reported on the birth certificate, or if it was coded as such on the inpatient hospital discharge records or during the prenatal period. The prenatal period was defined by the date of delivery, gestational age of the infant at delivery, and included the year prior to conception in defining prepregnancy diabetes. For a diagnosis of diabetes during pregnancy based on the prenatal data alone two or more ICD-9-CM diagnostic codes indicative of diabetes were required in the medical record [14], [26]. Primary and secondary inpatient hospital and prenatal ICD-9-CM diagnosis codes used to define diabetes included those for prepregnancy diabetes (i.e., 25000–25092). and GDM (i.e., 64801–64802, 64881–64882). Further classification into having prepregnancy diabetes or GDM was based on evidence of prepregnancy diabetes from any source; hence, the prepregnancy diabetes classification overruled. Maternal prepregnancy BMI (in kg/m^2^) was calculated based on maternal height and self-reported pre-pregnancy weight which are reported on the birth certificate. Self-reported weight and height are systematically under-reported [27], [28] and prepregnancy weight is inherently self-reported, therefore, it was assumed that BMI was under-reported by 5%; a sensitivity analysis compares results at 0% and 10% under-report.

### Statistical Analysis

The goal of our statistical analysis was to develop a model for the risk of diabetes during pregnancy, due to either prepregnancy diabetes or GDM. We did this with two (nested) logistic regression models which use the data from live singleton births in SC to estimate the risk of diabetes by type (GDM or prepregnancy diabetes) as a function of age (15–44-years-old), race/ethnicity, and BMI (rounded to the nearest whole number). The first (top level) model estimated the aggregate risk of diabetes during pregnancy (DM), defined as GDM or prepregnancy diabetes. The second model (nested level) used only the subset of data which included women with diabetes in order to estimate the likelihood that the woman had diabetes prior to pregnancy given that she had diabetes during pregnancy. From these results we extract the risk of GDM. Both regression models use the same explanatory variables as described in Equation 1, *α* is the intercept, *β_1_*–*β_5_* are the coefficients corresponding to *Race*, *BMI*, *BMI^2^*, *Age*, *Age^2^*, and the random intercept *ε_i_* term allows for differences in individual response when predictors have the same value. Both models predict the probability (*p_i_*) that the individual has diabetes, during pregnancy (DM) in the case of the top level model and prepregnancy diabetes given DM in the case of the nested level. The equation describes the relative effect of each predictor on the woman’s risk of disease (DM, prepregnancy diabetes given DM). The quadratic terms in BMI and age are included to capture the non-linear trend we anticipate in the model for these variables. The analysis was performed in Statistical Analysis System (SAS) 9.2 using GLIMMIX procedure, which allows random effects.
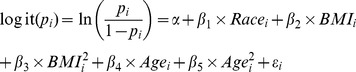
(1)


Model diagnostics and goodness of fit tests were performed using residual plots and observed-predicted value plots.

### Simulation Model

Our estimates of diabetes risk captured the mean response of an individual with a given set of characteristics. These results were then used to estimate the risk at a population level by assigning risk to simulated individuals. Simulation is an analytical tool used for evaluation of complex stochastic systems and for consideration of probable changes in those systems due to different sources of variation. In recent years, simulation has emerged as a powerful tool for health and economic evaluation [29], [30], and has been used to estimate the cost-effectiveness of treatment strategies for type 2 diabetes[31]–[33] and for screening practices for GDM [34].

We developed a simulation model of individuals in a population. We applied the predicted risk of diabetes during pregnancy to simulated individuals to estimate maternal diabetes prevalence at the population-level. First, 100,000 NHW and 100,000 NHB women were created whose ages conformed to the US Census distribution of age between 15–44-years-old. Second, race- and age-specific maternal BMI was also individually assigned based on estimates from NHANES. All analyses of NHANES data were conducted using the complex survey-specific procedures in SAS to account for the clustered sampling design and oversampling, and were adjusted for differential non-coverage and non-response across each NHANES cycle [12]. We tested the fit of the distribution of BMI and found that the lognormal and gamma distributions provided the best fit based on least mean squared error. We used the gamma distribution for BMI stratified by age, race and year; the shape and scale parameters of the gamma distribution were chosen such that the distribution’s mean and variance matched the mean and variance of the NHANES data for each year, race and age. Third, pregnancies were assigned to women according to race- and age-specific US natality data. Lastly, we simulated whether or not an individual developed diabetes. An individual’s risk of developing diabetes was based on age, race/ethnicity and BMI. Based on the two logistic regression models, women were first at risk of any type of diabetes during pregnancy (DM), then a portion of the women with diabetes were assigned prepregnancy diabetes. Simulation allows us to obtain different results for individuals with the same attributes and resulting risk level. Specifically, an individual’s risk of diabetes during pregnancy *p_i_* is determined by the logistic regression model (eq. 1), then we draw a random number between 0 and 1. If that random number is less than *p_i_* then the individual is said to have diabetes, if the random number is greater than *p_i_* then they are not diabetic. For example, if *p_i_* is 0.25 then any random number drawn between 0 and 0.25 will result is an individual being assigned diabetes, since random numbers are generated uniformly between 0 and 1, each individual will develop diabetes with probability *p_i_*, as is our goal. While the point estimate of the estimated risk (*p_i_*) is the same as the point estimate of the predicted risk, the standard error for the estimated risk (which accounts for uncertainty in the regression) is different than the standard error of the predicted risk (which accounts for both uncertainty in the regression and in the individual observation). Based on the sufficiently large sample size, the prediction standard error was approximated as the standard error for each estimate. Thus, the predicted values (and standard error) for every possible combination of model parameters were outputs of the regression models and are used to define the normal distribution of the risk of a woman developing diabetes used in the simulation.

In summary, variability in the model comes from two sources. First, race- and age-specific BMI is drawn from a distribution based on NHANES data. Second, the prediction error of the risk of diabetes (DM and GDM) estimated in the logistic regression model is normally distributed such that individuals with the same characteristics experience a different risk of diabetes during pregnancy. We used the same random number stream [35] for both NHB and NHW women when assigning age and BMI in order to remove variance attributable to different individual characteristics. We report diabetes prevalence in 5-year age groups between 15 and 44-years-old stratified by race/ethnicity for 1980, 1990, 2000, and 2008. The model was developed using Arena 13.5 and validated using the sample of SC women with known age, race, BMI, and diabetes statuses. The 95% CIs of the simulation estimates for diabetes prevalence (i.e., any diabetes, GDM, prepregnancy diabetes) covered the actual prevalence of the sample for both NHB and NHW women. Table 2 provides descriptive statistics of the SC sample. Simulated results used 40 replications; this was decided based on a desired half-width of <.2% for the overall prevalence estimates. See [36] for information on selecting number of replications. Rounding was done to the nearest decimal and results in CIs that appear to be of 0% width. Rounding may impact point estimates reported in results.

**Table 2:** SC population^a^ characteristics and simulation validation.

A. Characteristics of pregnant women in SC		
	Mean age	Mean BMI^b^ (kg/m^2^)
NHW	27.1	27.5
NHB	24.7	30.4
**B. Simulation validation**		
	**% of pregnant women with diabetes**	
	**Actual**	**Simulated (95% CI)**
Diabetes		
NHW	8.0	8.1 (8.0, 8.1)
NHB	8.9	9.0 (8.9, 9.1)
Prepregnancy DM		
NHW	1.7	1.7 (1.7, 1.8)
NHB	2.9	2.9 (2.9, 3.0)
GDM		
NHW	6.3	6.4 (6.3, 6.4)
NHB	6.0	6.1 (6.0, 6.1)

Abbreviations: CI, confidence interval; DM, diabetes mellitus; PPDM, prepregnancy diabetes mellitus; GDM, gestational diabetes mellitus; NHW, non-Hispanic white; NHB, non-Hispanic black.

a Pregnant NHW and NHB women ages 15–44 in SC from January 2004-December 2008.

b Assumes that prepregnancy BMI was underreported by 5% on the birth certificate.

## Results

The regression predictor estimates (and 95% confidence intervals) for diabetes and prepregnancy diabetes risk (given DM) are provided in Table 3. These are then used in the simulation model, as discussed in the methods section, to obtain our national estimates. Validation of our simulation approach is provided in Table 2B. This shows that the methodology of using our statistical model applied to a simulated population accurately predicts outcomes for that population. This is not a validation of the regression model itself, but rather of the approach.

**Table 3:** Logistic regression equation predictor estimates.

Predictor	Diabetes		Prepregnancy Diabetes	
	Estimate	95% CI	Estimate	95% CI
Intercept	−8.5440	−8.8939, −8.1941	−1.2150	−1.9449, −0.4850
Race, NHB	0.0500	0.0180, 0.0819	0.4614	0.3951, 0.5277
BMI, kg/m^2^	0.1552	0.1446, 0.1658	0.0407	0.0198, 0.0617
BMI^2^, kg/m^2^	−0.0013	−0.0014, −0.0011	−0.0002	−0.0005, 0.0001
Age	0.1230	0.1017, 0.1444	−0.0753	−0.1212, −0.0293
Age^2^	−0.0009	−0.0013, −0.0005	0.0011	0.0004, 0.0019

Results assume BMI is underreported by 5%.

From 1980 through 2008, in NHW the estimated prevalence of GDM increased 2.7% (95% CI 2.6%–2.8%) (Table 4). All prevalence estimates are reported to the single decimal point so CIs that appear to have a width of 0% are due to rounding. In NHB the estimated prevalence of GDM increased 2.5% (95% CI 2.4%–2.5%). The increase in GDM prevalence over time was higher in NHW than NHB women resulting in higher estimates of GDM prevalence subsequent to 1980 in NHW as compared to NHB women. In NHW women the estimated prevalence of prepregnancy diabetes increased from 1.0% (95% CI 0.9%–1.0%) in 1980 to 1.9% (95% CI 1.8%–1.9%) in 2008. In NHB women from 1980 through 2008 the estimated prevalence of prepregnancy diabetes increased from 1.7% (95% CI 1.6%–1.7%) to 3.2% (95% CI 3.1%–3.2%). Prepregnancy diabetes prevalence estimates were higher in NHB than NHW women in 1980 through 2008, with differences increasing over time (see Table 4). Combining these changes resulted in an overall increase in the estimated diabetes prevalence during pregnancy of 3.6% (95% CI 3.5%–3.7%) in NHW and 4.0% (95% CI 3.9%–4.1%) in NHB women.

**Table 4:** US population simulation estimates of diabetes prevalence (95% CI) during pregnancy in non-Hispanic white and non-Hispanic black women ages 15–44.

	non-Hispanic white				non-Hispanic black			
	1980	1990	2000	2008	1980	1990	2000	2008
**MAIN ANALYSIS**								
**5% BMI under-report**								
Diabetes								
Total	5.1(5.0, 5.2)	6.5(6.4, 6.6)	8.1(8.0, 8.2)	8.7(8.5, 8.8)	5.6(5.6, 5.7)	7.1(7.1, 7.2)	8.9(8.8, 9.0)	9.6(9.5, 9.7)
Ages 15–19	2.2 (2.0, 2.3)	2.6(2.4, 2.7)	2.8(2.6, 3.1)	2.8(2.5, 3.0)	2.6(2.5, 2.7)	3.2 (3.1, 3.3)	3.7(3.6, 3.8)	4.1(3.9, 4.2)
Ages 20–24	3.4(3.3, 3.6)	3.8(3.7, 4.0)	4.9(4.7, 5.1)	5.0(4.8, 5.2)	4.2(4.1, 4.3)	5.0(4.9, 5.1)	6.3(6.1, 6.4)	6.7(6.5, 7.0)
Ages 25–29	5.5(5.3, 5.7)	5.9(5.7, 6.1)	7.1(6.9, 7.3)	7.8(7.6, 8.0)	7.0(6.9, 7.2)	7.9(7.8, 8.1)	9.8(9.6, 10.0)	10.0(9.8, 10.2)
Ages 30–34	8.1(7.9, 8.4)	9.1(8.8, 9.3)	10.0(9.8, 10.2)	10.9(10.7, 11.1)	10.7(10.5, 11.0)	12.4(12.1,12.7)	14.2(13.9, 14.5)	14.3(14.0, 14.6)
Ages 35–39	11.0(10.4, 11.5)	13.0(12.5, 13.4)	13.8(13.5, 14.1)	14.1(13.6,14.6)	14.7(14.1, 15.3)	16.1(15.6, 16.6)	16.7(16.2, 17.3)	18.5(17.9, 19.1)
Ages 40–44	15.2(13.2, 17.2)	14.9(13.5, 16.3)	16.4(15.2, 17.6)	18.6(17.3, 19.8)	18.4(16.6, 20.2)	20.6(19.1, 22.1)	24.1(22.1, 26.1)	21.3(20.0, 22.5)
Prepregnancy diabetes	1.0(.9, 1.0)	1.3 (1.3, 1.3)	1.7(1.6, 1.7)	1.9(1.8, 1.9)	1.7(1.6, 1.7)	2.2(2.1, 2.2)	2.9(2.8, 3.0)	3.2(3.1, 3.2)
GDM	4.1(4.0, 4.2)	5.2 (5.2, 5.3)	6.4(6.3, 6.5)	6.8(6.7, 6.9)	4.0(3.9, 4.0)	4.9(4.9, 5.0)	6.0(6.0, 6.1)	6.4(6.3, 6.5)
**SENSITIVITY ANALYSIS**								
**0% BMI under-report**								
Diabetes	5.6 (5.5, 5.7)	7.2 (7.1, 7.3)	8.9 (8.8, 9.0)	9.5 (9.4, 9.6)	6.2 (6.1, 6.3)	7.8 (7.7, 7.9)	9.7 (9.6, 9.8)	10.4 (10.3,10.5)
Prepregnancy DM	1.1 (1.1, 1.1)	1.5 (1.5, 1.5)	1.9 (1.8, 2.0)	2.1 (2.0, 2.2)	1.9 (1.9, 1.9)	2.5 (2.4, 2.6)	3.2 (3.1, 3.3)	3.5 (3.4, 3.6)
GDM	4.5 (4.4, 4.6)	5.7 (5.6, 5.8)	7.0 (6.9, 7.1)	7.5 (7.4, 7.6)	4.3 (4.2, 4.4)	5.3 (5.2, 5.4)	6.5 (6.4, 6.6)	6.9 (6.8, 7.0)
**10% BMI under-report**								
Diabetes	4.6 (4.5, 4.7)	6.0 (5.9, 6.1)	7.4 (7.3, 7.5)	7.9 (7.8, 8.0)	5.1 (5.0, 5.2)	6.5 (6.4, 6.6)	8.1 (8.0, 8.2)	8.9 (8.8, 9.0)
Prepregnancy DM	0.8 (0.8, 0.8)	1.1 (1.1, 1.1)	1.5 (1.4, 1.6)	1.6 (1.6, 1.6)	1.5 (1.5, 1.5)	2.0 (2.0, 2.0)	2.5 (2.4, 2.6)	2.9 (2.8, 3.0)
GDM	3.8 (3.7, 3.9)	4.8 (4.7, 4.9)	5.9 (5.8, 6.0)	6.3 (6.2, 6.4)	3.7 (3.6, 3.8)	4.5 (4.4, 4.6)	5.6 (5.5, 5.7)	6.0 (5.9, 6.1)

The estimated diabetes prevalence during pregnancy increased between 1980 and 2008 within each age group in NHW women. However, the estimated prevalence increased in younger NHB women, but leveled off between 2000 and 2008 in NHB women between 30–34 and 40–44 years-old (Table 4). For example, prevalence estimate for women ages 40–44 in 2008 was 18.6% (95% CI 17.3%–19.8) in NHW and 21.3% (95% CI 20.0%–22.5%) in NHB. In NHW women the prevalence increase was highest in women 40–44 (3.33%, 95% CI 1.3%–5.4%) and lowest in those ages15–19 (0.6%, 95% CI 0.4%–0.9%). For NHB women, the increase in prevalence was highest in women 35–39 (3.8%, 95% CI 3.1%–4.5%) and lowest in those ages 15–19 (1.5%, 95% CI 1.3%–1.6%).

**Figure 2:**
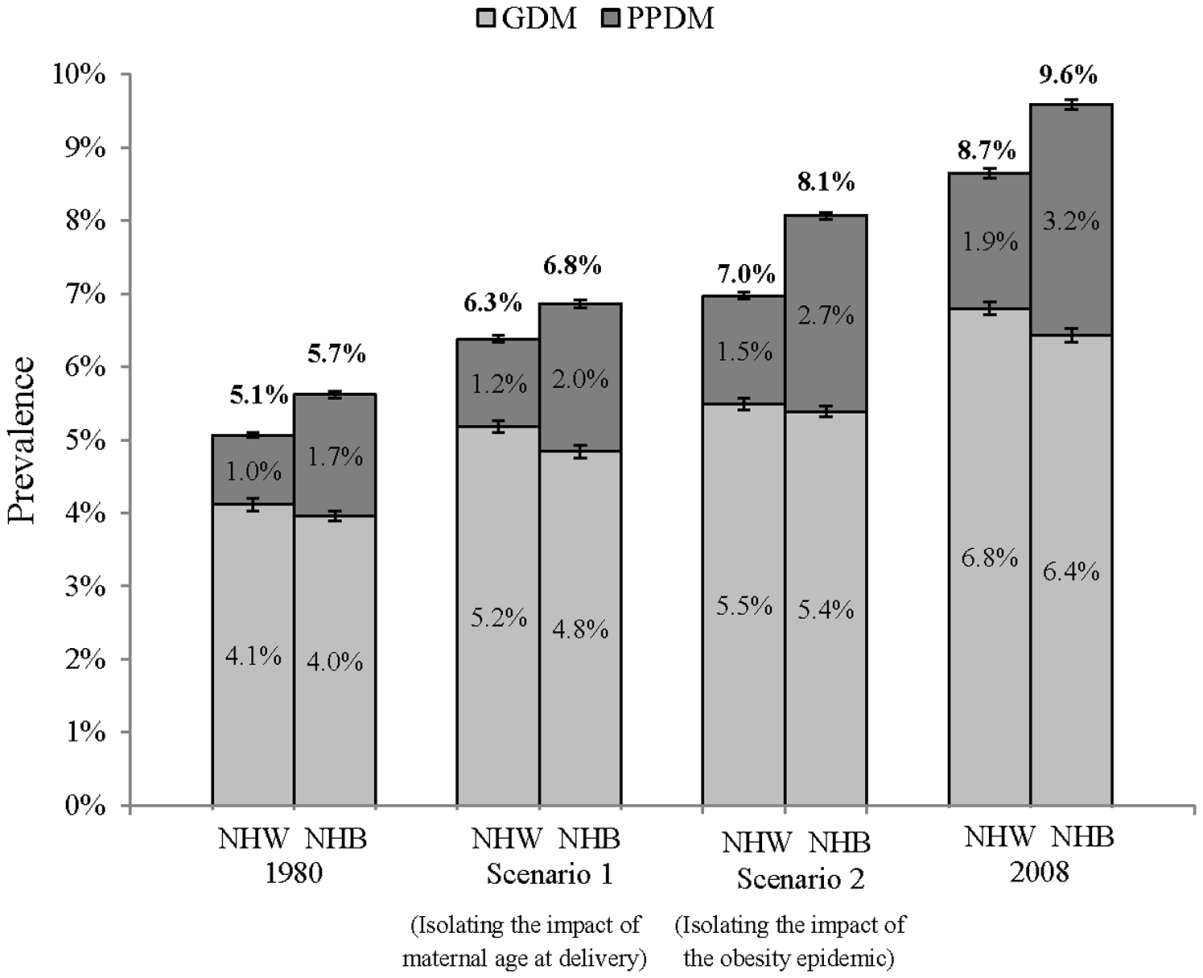
Diabetes prevalence estimates in NHW and NHB pregnant women ages 15–44 in Scenarios 1 & 2. Scenario 1 employed the 2008 US age and natality structure and 1980 BMI estimates (by age and race). In Scenario 2, the 1980 US age and natality structure was used with the 2008 BMI estimates.

The potential impact of increases in maternal age at delivery on diabetes prevalence during pregnancy from 1980–2008 was evaluated (Figure 2, Scenario 1; 2008 prevalence given increased maternal age at birth without the obesity epidemic). The projections indicate that the prevalence of GDM in 2008 would have been 5.2% (95% CI 5.1%–5.2%) representing a signficant increase over prevalence estimates in 1980 [4.1% (95% CI 4.0%–4.2%)]. The projections also indicate that the prevalence of prepregnancy diabetes in 2008 would have been 1.2% (95% CI 1.2%–1.3%) representing a significant increase over prevalence estimates in 1980 [1.0% (95% CI 0.9%–1.0%)]. Results were similar in NHB.

Similarly, the potential impact of increases in maternal BMI on diabetes prevalence during pregnancy from 1980–2008 was also evaluated (Figure 2, Scenario 2; 2008 prevalence given increased maternal BMI without increased maternal age at delivery). The projections indicate that the prevalence of GDM in 2008 would have been 5.5% (95% CI 5.4%–5.6%) which represents a signficant increase over prevalence estimates in 1980 [4.1% (95% CI 4.0%–4.2%)]. The projections also indicate that the prevalence of prepregnancy diabetes in 2008 would have been 1.5% (95% CI 1.4%–1.5%) which represents a signficant increase over prevalence estimates in 1980 [1.0% (95% CI 0.9%–1.0%)]. Results were similar in NHB.

Finally, we completed a sensitivity analysis to examine the impact of under-reporting prepregnancy weight on the birth certificate (see Table 4) at 0%, 5% (base case), and 10% BMI under-report levels. Diabetes prevalence estimates (total and by type) in NHW and NHB women were statistically higher when BMI is assumed to be reported correctly and statistically lower when BMI is assumed to be under-reported by 10% (Table 4).

## Discussion

This study examined the impact of changes in maternal age and prepregnancy BMI on the prevalence of GDM and prepregnancy diabetes over time at the national level in NHW and NHB women. From 1980–2008, simulation estimates of diabetes prevalence in pregnant NHW and NHB women increase, with higher increases over time in diabetes prevalence in NHB than NHW women. Interestingly, at each time point the higher diabetes prevalence during pregnancy in NHBs resulted solely from higher levels of prepregnancy diabetes with GDM prevalence levels actually being lower in NHB than NHW women subsequent to 1980.

Our national prevalence estimates for prepregnancy diabetes and GDM follow the same pattern, but are slightly higher than results from a study in Southern California that reports 2005 prevalence estimates for prepregnancy diabetes of 1.5% in NHW and 2.6% in NHB, and prevalence estimates for GDM of 5.3% in NHW and 5.0% in NHB [4]. In contrast, our national prevalence estimates are much higher, but follow similar increasing trends as a national study based solely on hospital discharge data that reported diabetes prevalence during pregnancy (i.e., prepregnancy and GDM combined) increased from 3.49% in 1994 to 5.47% in 2004 [37]. Similarly, a study based on National Hospital Discharge Survey’s reported the prevalence of GDM increased from 2.0% to 3.6% in white women and from 1.5% to 4.1% in black women from 1989 to 2004 [38].

One limitation of our study is the use of administrative databases and the reliability of data obtained from these databases. Data from all births in SC between 2004 and 2008 were used to assign the risk of GDM and prepregnancy diabetes. Hence, any errors or bias in the assignment of risk, based on misclassification of maternal diabetes status, age, race or prepregnancy BMI within the SC data were propagated to the national level. We used data from three sources to define diabetes status in SC, namely the birth certificate, and medical records from delivery and prenatal care. Moreover, in 2004 the South Carolina birth certificate was revised to improve the quality of the data: check boxes were added to differentiate between gestational and established diabetes; and information on maternal height and pre-pregnancy weight was added. A validation study of a comparable birth certificate was conducted on a population-based sample of 4,541 live births in Washington State [39]. True positive fraction combining information across the birth certificate and hospital discharge data and using medical record review as the gold standard was 93.3 (95% CI 86.9–99.7) for GDM and 96.9 (95% CI 91.6–100) for prepregnancy diabetes [39]. Respective false positive fractions were 0.9 (95% CI 0.5–1.4) and 0.5 (95% CI 0–1.1) [39]. Previous studies have validated the reliability of maternal BMI from birth certificates [40]–[42], with high correlation between self-report and clinically measured pre-pregnancy BMI that do not seem to differ by race/ethnicity, gestational age, or weight itself [43]. To overcome the limitation of potential incorrect reporting of prepregnancy BMI, a sensitivity analysis assuming underreporting of maternal BMI by 0%, 5% or 10% was also conducted. Another limitation, separate from the validity of the SC data is the generalizability of the SC data to the national level. Importantly, the model developed using SC data was based only on the relationship between maternal pre-pregnancy BMI, age and race and their association with diabetes status (i.e., prepregnancy or GDM). Hence, to the extent that the relationship between these factors (maternal BMI, age and race) and maternal diabetes is consistent across different environments (i.e., dietary patterns, socioeconomic status and geographic locations) the model should hold. Ideally, we would have validated the SC regression model using data available from other states, or even national data; however, unfortunately equivalent national data was not available nor was it feasible within the time frame of the study to obtain data from even one other state with similar resources (i.e., fewer than a handful of states have resources similar to SC in their ability to interlink required data sources). A final study limitation is that the criteria for diagnosing GDM were revised in 2011 based on results from the Hyperglycemia and Adverse Pregnancy Outcomes study [44]; however, our study is unable to address the impact of this change on the projected diabetes prevalence during pregnancy.

A strength of our approach is that risk was assigned uniformly over time and varied only due to changes in the race/ethnicity-specific maternal age, BMI and natality structure of the population; hence, it was possible to examine the potential impact of each of these items on diabetes prevalence during pregnancy from 1980 through 2008. Furthermore we were able to examine trends over time without the effects of changes in definition, screening, or awareness.

The results of this study indicate that increased maternal age and the obesity epidemic both contribute substantially to the increasing prevalence of GDM as well as prepregnancy diabetes. Racial disparities in diabetes prevalence during pregnancy appear to stem from a higher prevalence of prepregnancy diabetes in NHB than NHW women, with slightly lower prevalence estimates for GDM in NHB than NHW women. The increasing prevalence of prepregnancy diabetes is disconcerting given that diagnosis of diabetes at a younger age results in greater duration, co-morbidity burden and earlier mortality [45]. Exposure to maternal diabetes early in pregnancy is associated with birth defects, high birth weight, increased childhood and adult obesity and increased risk of type 2 diabetes later in pregnancy [12], [13], [15], [18], [46]. Interventions are required that increase the awareness and control of diabetes prior to pregnancy and prevent the development to type 2 diabetes following GDM.
